# Repeatability of circadian behavioural variation revealed in free-ranging marine fish

**DOI:** 10.1098/rsos.160791

**Published:** 2017-02-15

**Authors:** Josep Alós, Martina Martorell-Barceló, Andrea Campos-Candela

**Affiliations:** Instituto Mediterráneo de Estudios Avanzados, IMEDEA (CSIC-UIB), C/ Miquel Marqués 21, 07190 Esporles, Illes Balears, Spain

**Keywords:** acoustic tracking, circadian clocks, behavioural syndrome, chronotypes, hidden Markov models, repeatability

## Abstract

Repeatable between-individual differences in the behavioural manifestation of underlying circadian rhythms determine chronotypes in humans and terrestrial animals. Here, we have repeatedly measured three circadian behaviours, awakening time, rest onset and rest duration, in the free-ranging pearly razorfish, *Xyrithchys novacula*, facilitated by acoustic tracking technology and hidden Markov models. In addition, daily travelled distance, a standard measure of daily activity as fish personality trait, was repeatedly assessed using a State-Space Model. We have decomposed the variance of these four behavioural traits using linear mixed models and estimated repeatability scores (*R*) while controlling for environmental co-variates: year of experimentation, spatial location of the activity, fish size and gender and their interactions. Between- and within-individual variance decomposition revealed significant *R*s in all traits suggesting high predictability of individual circadian behavioural variation and the existence of chronotypes. The decomposition of the correlations among chronotypes and the personality trait studied here into between- and within-individual correlations did not reveal any significant correlation at between-individual level. We therefore propose circadian behavioural variation as an independent axis of the fish personality, and the study of chronotypes and their consequences as a novel dimension in understanding within-species fish behavioural diversity.

## 1. Introduction

Humans show consistent and repeatable between-individual differences in their average level of circadian behaviours, such as awakening time, rest onset or rest duration, defining different chronotypes [1,2]. Chronotypes reflect the behavioural manifestation of an individual's underlying molecular clock, have fundamental implications for wellbeing and health and have been frequently linked to fitness ([3] and see review by Adan *et al*. [4]). The existence of chronotypes in free-ranging animals is poorly studied [5,6], but the emerging empirical and theoretical approaches supporting the existence and maintenance of between-individual variation in behavioural and personality traits, such as boldness, aggressiveness, activity, exploration and sociability [7–9], suggest that chronotypes could be present and be widespread across taxa [10].

Several authors have studied circadian behavioural variation in free-ranging animals (see review by Randler [10]), including fish [11–13]. However, only three studies have measured repeatability scores (*R* [14]) as a standard measure to statistically assess behavioural consistency and fulfil the criterion of chronotype [15–17]. While Stuber *et al*. [16] found low *R*s in circadian-related behaviours in great tits, *Parus major*, Stuber *et al*. [17] and Steinmeyer *et al*. [15] found consistent between-individual differences and high *R*s in great tits exposed to predation risk and blue tits, *Cynistes caeruleus*, respectively. These recent evidences of chronotypes in terrestrial animals have provided a novel dimension in relation to chronobiology to understand individual behavioural diversity and many ecological and evolutionary processes [10,18]. As regards aquatic animals, and particularly fishes, the existence of chronotypes (i.e. the assessment of the repeatability in traits like a wakening time, rest onset or rest duration) has never been explored in the wild.

Most fish species show a circadian behaviour (active/resting cycle) governed by molecular clocks that is consistent with the mammalian-like sleep architecture [11,19,20]. With the recent development of biotelemetry, ecologists have gained a powerful tool to study between-individual behavioural variation in the aquatic system [21], especially regarding the identification of the active/resting cycle in free-ranging fish [22,23]. Furthermore, several studies have recently demonstrated high repeatability of the spatial behavioural variation in free-ranging fish [24–27]. Additionally, the development of robust statistical tools such as hidden Markov Models (HMMs) [28] and state-space models (SSMs) [29] have notably facilitated the identification of behavioural states (resting/active) from telemetry data and offer now the tools to properly develop individual-based chronobiological studies in free-ranging fish.

The main objective of this work was to assess the existence of chronotypes or consistent between-individual differences in mean circadian behavioural traits in a marine free-ranging fish. We first identified using linear mixed models (LMM) several environmental covariates that may affect three circadian-related behaviours and one fish personality trait (daily activity) measured in the field using acoustic tracking. Subsequently, we decomposed the variance of these four traits into between- and within-individual sources and estimated *R* while controlling for previous environmental covariates. Behavioural syndromes (i.e. a suite of correlated repeatable traits) have now been widely described in the literature across taxa [30], even in fish [31]. Our final objective was therefore to explore whether chronotypes form part of the personality architecture or can be considered as an independent axis of the animal personality by decomposing the correlations among repeatable traits into between- and within-individual correlations.

## 2. Material and methods

### 2.1. Species case-study and acoustic tracking experiment

**Figure 1.**
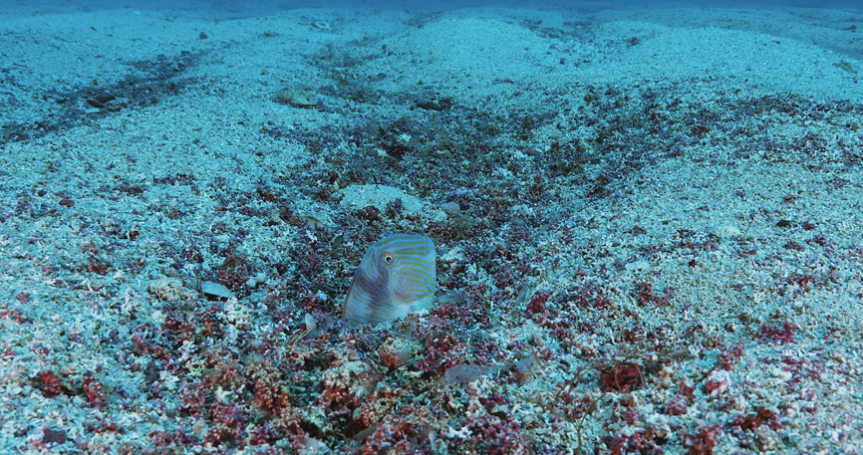
The pearly razorfish, *Xyrithchys novacula*, is a small-bodied labrid widely distributed in temperate areas that buries itself in the sand during the night-time to rest and avoid predators. Our study reveals that individual heterogeneity in awakening time, rest onset or rest duration is highly repeatable and predictable and conforms to chronotypes. This individual-based circadian behavioural variation can be considered as an independent axis of the fish personality.

**Figure 2.**
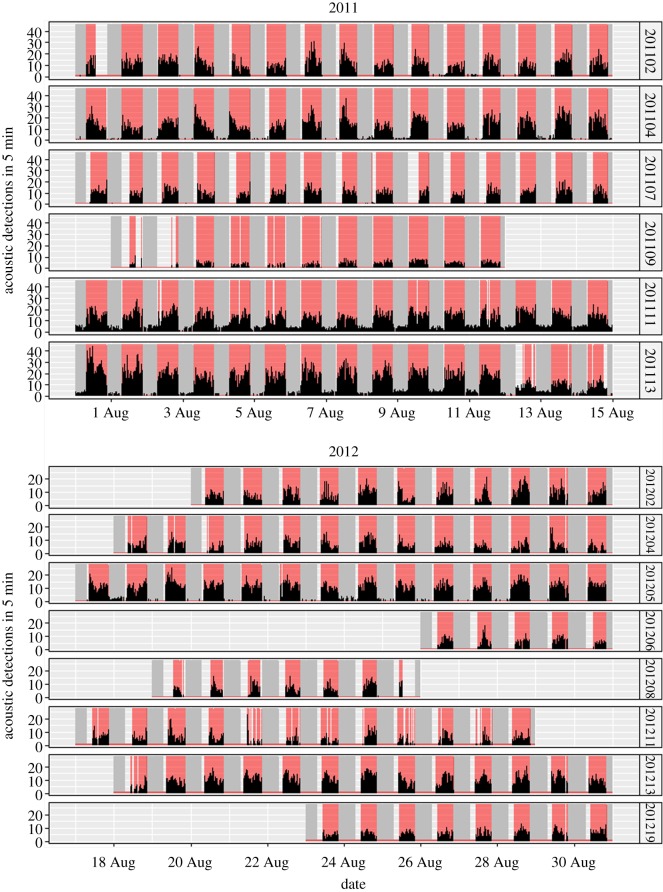
Markovian chains (sequences of time every 5 min) of acoustic detections obtained from different individuals of free-ranging pearly razorfish, *Xyrithchys novacula*, acoustically tracked in our study. The identification code of the individual is shown vertically and grey bands refer to night-time periods according to the local sunrise and sunset data. The red bands show the predicted state (active) at any moment by our HMM. Note how the predicted active state fits with the period with higher acoustic detections suggesting the individuals are outside the sand. Also note how some individuals still have detection during the night-time (although the HMM clearly identifies the two states), as probably they buried close to a receiver and some signals were still received.

The pearly razorfish, *Xyrithchys novacula* (figure 1), is a small-bodied labrid widely distributed in soft shallow habitats of temperate areas [22]. During the night-time, it buries itself in the sand to rest and avoid predators; it is a diurnal species (see the electronic supplementary material, movie S1). The pearly razorfish can also occasionally bury during the daytime in the presence of diurnal predators such as dolphins, but usually remains over the sand foraging and feeding on small invertebrates and bivalves [32] (see the electronic supplementary material, movie S1). These clear circadian rhythms moving in and out of the sand (their refuge) according to the sunset and sunrise, revealed in this species using acoustic telemetry by Alós *et al*. [22] (figure 2), offered us a good opportunity to contextualize our working hypothesis of repeatable circadian behavioural variation.

In 2011 and 2012, a total of 21 free-ranging individuals were tracked using an array of acoustic receivers in a marine protected area (MPA) located in Palma Bay, northwest Mediterranean (39.44′ N 2.73′ E, electronic supplementary material, figure S1). The details of the tracking experiment are widely explained in Alós *et al.* [22]. Briefly, fish were tagged with a tiny acoustic tag (Sonotronics^©^ model PicoTag-2) that emitted every minute an individual acoustic signal that was recorded by a fixed array of 21 omni-directional acoustic receivers (Sonotronics^©^ model SUR-1; see the electronic supplementary material, figure S1). The miniaturized tag used measured 19 × 7.1 mm, weighed 1 g in water, and did not exceed 2% of the fish's body weight in any case. The array allowed for continuous tracking of the individual when it was foraging out of the sand (i.e. when the individual remained buried during night-time, the detection range is dramatically reduced due to the physical barrier formed by the sand, see [22]).

According to the limited movement of the pearly razorfish and the movement path (see below), the fish remained within our detection range all the time until loss of battery power (up to 23 days according to Alós *et al*. [22]). However, the pearly razorfish has a period of some days of abnormal behaviour after tagging, during which fish remained buried in the sand [22]: these data were discarded. After this initial period, individually assessed using continuous wavelet transformations according to Alós *et al.* [22], the effects of the tag in the fish are negligible and have a very limited impact on their survival [27]. After cleaning the detection time-series following the decision criteria tree depending on false (i.e. environmental noise) and spurious (i.e. isolated detection in a 24 h period) detections for discarding mortalities proposed by March *et al*. [23], the dataset for testing our hypothesis of the existence of chronotypes was composed of 14 time-series of acoustic detections (six individuals in 2011 and eight individuals in 2012) tracked for up to 14 days (total of *n* = 148 observation days; electronic supplementary material, table S1 and figure 2).

### 2.2. Determination of daily circadian-related behaviours using hidden Markov models and state-space models

We used an HMM to automatically classify different behavioural states (active (A) versus resting (R), figure 2) in each fish using the acoustic tracking data generated in our experiment. The HMM developed here assumed that the true state constituted a hidden (unobserved) Markovian state variable that can be estimated from the observed pattern of acoustic detection events according to a switching 2 × 2 probability transition matrix (A → A, A → R, R → R, R → A). Therefore, we first constructed a sequence of acoustic detections pooled in bins of 5 min (Markovian chain) for each fish which constituted the input data for the HMM (figure 2). Five minutes ensured a good resolution to detect the awakening time, the rest onset and a proper identification of both discrete states (A versus R) using the HMM as revealed by the observed and predicted patterns shown in figure 2. We fitted using the expectation-maximization algorithm one HMM to each fish using the *depmix* function of the library depmixS4 [33] developed in R [34]. We restricted the number of states to two (A versus R) and considered Poisson-distributed data. For each fish, we used the predicted sequence of the active state to calculate on a daily basis: (i) the awakening time relative to sunrise (min) as the first bin for daily active behaviour, (ii) the rest onset relative to sunset (min) as the last bin for daily active behaviour, and (iii) the rest duration (hours) as the time difference between the rest onset of the previous day and the awakening time of the current day. We used the local daily sunset and sunrise data.

We also considered (iv) daily total travelled distance (m) calculated from the positions of tracked fish as a standard personality trait related to the activity of fish [31]. The extraction of positional data from conventional acoustic tracking is, however, not trivial as fish positions constitute a hidden state subjected to positioning error. SSMs are particular cases of HMM increasingly used to solve these issues in studies involving biotelemetry-based positional data [29], and here we considered a recent SSM implementation for the specific case of the pearly razorfish and acoustic tracking [35]. Briefly, the SSM developed by Alós *et al*. [35] allowed us to accurately estimate positional data at 15 min time-steps and the movement parameters that lead to the establishment of a home range area (defined as the area used by the pearly razorfish during its normal activities [35]) by combining two models: (i) a fish movement model (a specific case of random walk weighted by an Ornstein–Uhlenbeck process, with parameters: latitude and longitude of the centre, size and exploration rate of the home range [35]) and (ii) an observational model that considers environmental-related probability of detection. Once positions were estimated for each fish, daily total travelled distances were then calculated by the summation of all Euclidean distances between all two-consecutive positions of each day.

### 2.3. Repeatability of circadian behavioural variation

The repeatability score (*R*) represents the phenotypic variation attributable to individual heterogeneity and is often used to characterize animal personalities and detect behavioural types [14,36], or in our case chronotypes. *R* was estimated here as the quotient between the between-individual variance (the variance across random intercepts of individuals, *V*_ind0_) and the sum of *V*_ind0_ and the within-individual or residual variance (the variance associated with measurement error and phenotypic flexibility, *V*_e0_) for a given behavioural trait [14,36]. We fitted LMMs using the library MCMCglmm [37] of the R-package according to Harrison *et al*. [24] and Dingemanse & Dochtermann [36] to properly decompose the raw phenotypic variance into between- and within-individual variances. Several covariates that could potentially affect circadian behavioural variation were considered including individual-related variables (fish size and sex), and spatial (depth, habitat, latitude and longitude) and temporal (year) environmental variables.

We first assessed the existence of correlations between covariates in order to include in the data analysis uncorrelated variables and avoid colinearity issues. The pearly razorfish is a protogynous hermaphrodite and, in consequence, fish length was strongly correlated with sex (ANOVA: *F*-value = 18.95, *p*-value ≤ 0.001). We selected sex to be incorporated in the statistical analysis as sexual differences have been observed in other taxa [10,15], but significant effects were interpreted jointly (sex–fish size effect). We also considered the latitude and longitude of the centre of the home range estimated by our SSM approach described above [35]. Latitude and habitat (grain size) were highly correlated (linear model (LM): *t*-value = −25.61, *p*-value ≤ 0.001; see the electronic supplementary material, figure S1), and longitude and depth (m) were also highly correlated (LM: *t*-value = −18.52, *p*-value ≤ 0.001; see the electronic supplementary material, figure S1). We selected latitude and longitude of the centre of the home range to be incorporated in the model (as they provide information not only about the habitat and water depth but also other variables not sampled that covaried spatially) to avoid colinearity issues, but significant effects were discussed jointly (latitude–habitat and longitude–depth). Nevertheless, the results of the LMMs fitted with both sets of variables are presented in the paper (see Results). Two independent experiments were carried out in 2011 and 2012 and year was included as categorical variable.

We therefore fitted four different LMMs (one for each behavioural trait) to the following fixed effects: sex, latitude and longitude of the centre of the home range and year, and all two-order interactions (all continuous variables were mean centred), considering the identification of the fish (id) as random intercepts. The parameters and their Bayesian credibility intervals (BCI, 2.5% and 97.5%) of the LMMs were estimated using a Bayesian approach with the default settings of the library MCMCglmm. In all cases, convergence of the chains was attained and checked by plotting the MCMCglmm objects generated [37]. The full models (with all fixed effects) were reduced using bidirectional elimination (i.e. ‘step-by-step’ backward reduction, which is a combination of the backward and forward stepwise selection) until the lowest deviance information criterion (DIC) was attained.

We used the reduced LMM to compute adjusted-*R* scores for each trait (adjusted repeatability after controlling the confounding fixed effects [14]). The BCI of all adjusted-*R* values were interpreted to detect the presence of chronotypes, and a likelihood ratio test (LRT) was used to calculate the significance of the adjusted-*R* [14,36]. According to the LRT, the reduction in the DIC (ΔDIC) provided by the LMM where *V*_ind0_ was constrained to 0 was used to detect significant *V*_ind0_ [36]. DIC reductions by the unconstrained LLM compared to the constrained LLM larger than 2 were considered significant adjusted-*R*s. Temporal auto-correlation or time-dependence of the behavioural measures may lead to pseudo-*R* estimates and over-inflation of the *R* scores [36,38]. The Durbin–Watson (DW) statistic applied to the scaled residuals of each LMM was used to test temporal auto-correlation or pseudo-*R* scores in the circadian behaviours considered here. In all cases, alternative hypothesis (true temporal auto-correlation was greater than 0) was rejected (awakening time (DW = 2.1, *p*-value = 0.6891), rest onset (DW = 2.1, *p*-value = 0.78), rest duration and temporal auto-correlation discarded (DW = 2.08, *p*-value = 0.69) and daily travelled distance (DW = 2.19, *p*-value = 0.8946)).

### 2.4. Between-individual correlations among circadian behaviours and activity

We used bivariate (paired traits) LMM to decompose phenotypic correlations among paired circadian-related and personality traits (*r*_P_) into between- (*r*_ind_) and within-individual or residual (*r*_e_) correlations according to procedures described in Dingemanse & Dochtermann [36] to detect the existence of circadian behavioural syndromes. Although *r*_P_ has been traditionally used for detecting behavioural syndromes, recently it has been analytically described that only *r*_ind_ certainly describes true ones [39–41]. We therefore considered significant *r*_ind_ to be representative of behavioural syndromes. We used the MCMCglmm library to fit bivariate LMMs (six different models result of combining the four previously defined behaviours in pairs) with a weak inverse-Wishart prior structure with scalar parameters *V* = an identity matrix of size 2 and *ν* = 1.002 as suggested by Dingemanse & Dochtermann [36]. All fixed effects that remained in the reduced LMMs of the previous section were included in the models as covariates. To ensure convergence of the models, we considered 1 300 000 iterations, thinned every 1000 (thin) to avoid auto-correlation, and the first 300 000 were discarded (burning). The reductions in the DIC (ΔDIC), provided by the paired LMM where between-individual and residual covariance was constrained to 0, was used to detect significant correlation coefficients (DIC reductions larger than 2 were considered significant [24]). All models were fitted considering a Gaussian response, and rest duration and daily travelled distance were log-transformed to reach normality in the distribution of the residuals against covariates.

## 3. Results

**Figure 3.**
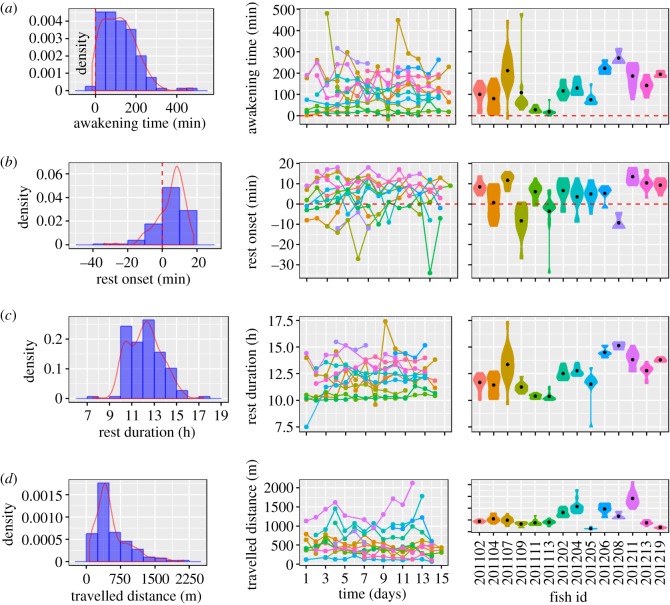
Density population plots (left column), daily individual values (middle column) and daily individual density plots (violin plots in right column) in (*a*) awakening time relative to sunrise (denoted by a dashed red line), (*b*) rest onset related to sunset (denoted by a dashed red line), (*c*) rest duration and (*d*) daily travelled distance obtained in the individuals of free-ranging pearly razorfish, *Xyrithchys novacula*, acoustically tracked in our study.

The mean and s.d. of awakening time (min relative to sunrise) and rest onset (min relative to sunset) were 117.9 ± 85.1 min and 5 ± 8.1 min, respectively (figure 3). Overall rest duration was 12.2 ± 1.5 h and individuals tracked travelled a daily distance of 554.1 ± 374.4 m, with a maximum observed distance of 2127.8 m (figure 3). Mean and s.d. values for the individuals are shown in the electronic supplementary material, table S1. The analysis of the potential environmental covariates affecting awakening time and rest duration suggested that both of them were affected (considering the BCI of the estimated parameters) by the latitude of the centre of the home range and the year of experimentation (table 1). The rest onset was only affected by the longitude of the centre of the home range suggesting that individuals living in higher longitudes had a delayed rest onset (table 1). Finally, the LMM fitted to the daily travelled distance only retained the latitude of the centre of the home range suggesting that larger travelled distances occurred in fish inhabiting lower latitudes (table 1). All parameters and BCI of the final models as well as DIC, *V*_ind0_ and *V*_e0_ are shown in table 1. The results of the LLMs considering fish size, depth and grain size instead of sex, and latitude and longitude of the centre of the home range, were essentially the same and are shown in the electronic supplementary material, table S2.

**Table 1.** Environmental covariates (posterior mean shown) and their Bayesian credibility intervals (lower (l-) and upper (u-) BCI) of the four linear mixed models (LMMs) fitted for the four behaviours studied here: awakening time relative to sunrise, rest onset relative to sunset, rest duration and daily travelled distance. The table shows the LMMs after the reduction according to the maximum explanatory power using the deviance information criterion (DIC). Latitude and longitude refer the position in UTM (zone 31S) of the centre of the home range as continuous variable, and year of experimentation was treated as categorical variable (estimate of 2012 shown with respect to 2011). The between- (*V*_ind0_) and within-individuals (*V*_e0_) variances as well as the adjusted repeatability (adjusted-*R*) for each trait are also shown. The DIC of the reduced LMM as well as the DIC of the constrained LMM (DICc) are shown for all behavioural traits.

	mean	l-BCI	u-BCI
awakening time (min)			
intercept	85.13	41.41	131.69
latitude (m)	−34.92	−71.19	−4.29
year (2012)	84.29	15.21	146.08
*V*_ind0_	2138.63	932.88	7161.87
*V*_e0_	3230.68	2571.37	4011.50
adjusted-*R*	0.40	0.27	0.64
DIC = 1751.96 (DICc = 1817.6)			
rest onset (min)			
intercept	4.90	1.76	7.57
longitude (m)	4.41	1.89	6.57
*V*_ind0_	13.49	5.72	45.81
*V*_e0_	32.17	24.97	38.80
adjusted-*R*	0.30	0.19	0.54
DIC = 1011.7 (DICc = 1071)			
log-rest duration (h)			
intercept	2.42	2.36	2.48
latitude (m)	−0.05	−0.09	−0.004
year (2012)	0.17	0.09	0.27
*V*_ind0_	0.01	0.002	0.01
*V*_e0_	0.01	0.004	0.01
adjusted-*R*	0.49	0.29	0.67
DIC = −338.14 (DICc = −269.5)			
log-travelled distance (m)			
intercept	6.15	5.75	6.49
latitude (m)	−0.28	−0.66	0.07
*V*_ind0_	0.33	0.16	0.90
*V*_e0_	0.07	0.06	0.09
adjusted-*R*	0.83	0.75	0.91
DIC = 41.6 (DICc = 310.3)			

Adjusted-*R* estimated for the four behavioural traits resulted in consistent and high predictability of between-individual differences (table 1). LRT test performed using the DIC of the unconstrained and constrained models suggested all estimated adjusted-*R*s were significant (table 1). The highest adjusted-*R* estimated (0.82 [0.72, 0.9]) corresponded to the standard personality trait defined by the daily travelled distance (figure 3). The adjusted-*R* scores for awakening time, rest onset and rest duration were smaller (0.4 [0.26, 0.64], 0.29 [0.19, 0.5] and 0.5 [0.3, 0.67], respectively) but in all cases the lower values of the BCI suggested the existence of significant chronotypes while controlling for the environmental covariates (figure 3 and table 1). Although we accepted the hypothesis of chronotypes, we did not find any significant phenotypic (*r*_P_), or between-individual (*r*_ind_) correlations among paired circadian-related and personality traits, and only a significant negative within-individual or residual correlation (*r*_e_) between awakening time and travelled distance was detected (table 2).

**Table 2.** Between- (*r*_ind_), within- (*r*_e_) and phenotypic correlations (*r*_P_) (plus Bayesian credibility interval, BCI) between the paired behaviours studied here: awakening time relative to sunrise (min), rest onset relative to sunset (min), rest duration (h, log-transformed) and daily travelled distance (m, log-transformed). Correlations in italics were assumed to be significant (ΔDIC between constrained and unconstrained model > 2).

	awakening time	rest onset	rest duration
*r*_ind_			
awakening time	—	—	—
rest onset	0.57 [−0.3, 0.93]	—	—
rest duration	0.4 [−0.37, 0.81]	−0.14 [−0.59, 0.76]	—
travelled distance	−0.03 [−0.68, 0.62]	0.34 [−0.47, 0.86]	0.31 [−0.52, 0.84]
*r*_e_			
awakening time	—	—	—
rest onset	−0.04 [−0.17, 0.16]	—	—
rest duration	0.07 [−0.11, 0.23]	0.03 [−0.16, 0.17]	—
travelled distance	*−0.37* [*−0.51, −0.24*]	−0.03 [−0.22, 0.1]	−0.07 [−0.2, 0.11]
*_r_*_P_			
awakening time	—	—	—
rest onset	0.25 [−0.2, 0.67]	—	—
rest duration	0.3 [−0.25, 0.7]	0.10 [−0.39, 0.65]	—
travelled distance	−0.11 [−0.58, 0.49]	0.22 [−0.34, 0.72]	0.19 [−0.36, 0.71]

## 4. Discussion

The study of the covariates affecting circadian behavioural variation in free-ranging pearly razorfish suggested that awakening time, rest duration and daily travelled distance were affected by the spatial location of the home range. With increasing latitude (which is negatively correlated with grain size of habitat), individuals significantly woke up earlier, had shorter rest duration and travelled less distance per day. Awakening time and rest duration were also significantly affected by the year of experimentation, and fish awoke later and rested for more time in 2012. Several studies have found how spatial and temporal variables can affect circadian-related behaviours [15,17]. The effect of the spatial and temporal dimension can be related to indirect effects of predation risk, temperature, light conditions or the reproductive season, or any other variable covarying with the variables considered here [15,17]. However, the exact mechanism behind the spatial and temporal relationships and circadian-related behaviours we found cannot be fully disentangled with our data (i.e. the latitude and longitude can be correlated with more variables not sampled here). It was relevant for the purpose of our study to consider the spatial and temporal dimensions as covariates to control for potential environmental influences when exploring the repeatability of the circadian behavioural variation.

Awakening time variation was much greater than rest onset suggesting a larger window to start the activity (i.e. awakening time range: (−16, 480) min and rest onset range: (−34, 18) min). The night-time can be considered as a dangerous environment for the pearly razorfish [22], and our findings suggested that sunset is a strong signal of perceived predation risk. In fact, longitude (correlated with depth) was the only significant predictor of rest onset. Thus, individuals inhabiting higher longitudes extended their daily activity by some minutes before resting, as darkness arrived later. Light intensity and predation risk are some of the most important environmental factors affecting circadian-related behaviours [10,15,17], and the findings obtained in the pearly razorfish suggest that these factors have to be considered when exploring chronotypes. We did not find a significant proportion of variance explained by sex and this factor was not retained in the final LMMs. This lack of significant variance could be attributed to our limited sample size but also because our study was focused outside the reproductive season. Further work increasing the number of tracked males and females and extending the tracking period into the reproductive season (May–June) should shed light upon a better picture of whether a sexual relationship in our behavioural traits exists as found in other taxa [10,15].

On average, the adjusted-*R*s for all traits were significant and relatively high (i.e. average adjusted-*R* = 0.5 [0.37, 0.69]) using an overall average of 10 observations per individual, suggesting the existence of chronotypes and reinforcing the idea of activity (as daily travelled distance) as a fish personality trait. Acoustic tracking and, in general, any telemetry study is usually constrained to limited sample sizes due to the high cost of the technology. Therefore, it is possible that our adjusted-*R* values were overestimated due to the relatively small sample size in our study. However, a recent meta-analysis on animal personality by Bell *et al.* [7] reported average *R* of 0.37 among 98 species across several taxa, which was estimated by on average 4.4 repeated observations of behaviours and sample sizes (number of individuals) ranging from 5 to 1318 individuals. Therefore, we feel it is a safe conclusion that circadian behavioural variation is repeatable in pearly razorfish. Our chronotypes can be categorized along three main behavioural axes. The first and second axes were formed by early-delayed risers and early-delayed rest onset individuals. The third axis determined a short-large rest duration gradient as there are individuals that consistently rest for more time than others. Finally, the daily travelled distance suggested the existence of short-large traveller individuals. Our measure of daily travelled distances is commonly used as a measure of activity in fish [31], and our findings support that activity is a strong fish personality-related trait with a high degree of behavioural predictability. In general, our adjusted-*R*s are similar and within the range of those reported by Stuber *et al*. [17] and Steinmeyer *et al*. [15] in great and blue tits, respectively, and support our initial hypothesis of the existence of repeatable circadian behavioural variation.

Repeatability in behaviours is a pre-requisite exploring the existence of behavioural syndromes [8,30,40]. Behavioural syndromes have been found in humans regarding their sleep behaviour, and perhaps morning ‘larks’ and evening ‘owls’ (positive between-individual correlation between awakening, rest onset and several personality traits) are the common examples [42]. Harrison *et al*. [24] has recently revealed the existence of a spatial-related behavioural syndrome determining ‘resident’ and ‘mobile’ fish using a similar biotelemetry study. The proper partitioning of the correlations among paired traits in the pearly razorfish has not, however, revealed a circadian behavioural syndrome. There was only one residual (within-individuals) significant negative correlation between awakening time and daily travelled distance that could be related to the daily rest requirements or hunger levels of the individual. This finding is consistent with the unique study that explored the existence of circadian behavioural syndromes in animals by Stuber *et al*. [16], who also failed to find sleep behavioural syndromes. We therefore propose that circadian behavioural variation forms an independent axis of the animal personality. Considering the number of observations and the repeatability scores in our traits, the power of our dataset should be high enough to detect behavioural syndromes, although larger sample sizes are recommended [36]. Further research for disentangling the existence of circadian behavioural syndromes should include not only an increase in the number of individuals tracked but also the integration of other personality-related traits not considered here, such as boldness, aggressiveness or sociability, into the analysis.

Our work has some limitations that should be considered in further analysis. Owing to the limited body size of the pearly razorfish, we needed very small acoustic tags (pico-tags), limiting notably our tracking and potentially affecting our *R* estimates [38]. Although our analysis discarded the existence of temporal auto-correlation that could lead to pseudo-*R*, we recommend extending the tracking experiments, not only on time (tracking days) and individuals but also across contexts (e.g. spawning season), to provide a better picture of chronotypes in free-ranging fish. Despite this limitation, we have provided the first evidence of repeatable circadian behavioural variation in fish in traits like awakening time, rest onset or rest duration, and some ecological and evolutionary consequences can be considered.

Fish balance the trade-off between foraging and exposures to predation to maximize their fitness and, therefore, predation could generate natural selection against some chronotypes [17]. Similarly, man is today a major selective pressure on animals through hunting and fishing [43]. The pearly razorfish is highly exploited and behaviourally selected by recreational fisheries. One would expect that those individuals with delayed awakening times would be less exposed to fishing pressure and the number of encounters with fishers would lead to selection gradients similar to those reported by the movement traits [27]. The relevance of selection (either natural or human-caused) acting on circadian-related traits relies on the genetic basis of chronotypes being high and well established [6,11,44,45]. Therefore, the genetic architecture of fish chronotypes would notably help ecologists to understand the evolution of resting in fish in response to natural and different sources of selection imposed by fishing. We therefore feel that the study of individual chronobiologies facilitated by new tracking technologies and statistical approaches opens up a novel dimension in understanding within-species behavioural diversity and its consequences.

## Supplementary Material

Figure S1 Map of the study area and array of acoustic omnidirectional receivers where individuals of free-ranging peraly razorfish, Xyrchthys novacula, were electronically tracked

## Supplementary Material

Movie S1 Underwater video of free-ranging pearly razorfish, Xyrithchys novacula in the waters of Mallorca Island

## Supplementary Material

Table S2 Environmental co-variates (posterior mean showed) and their Bayesian Credibility Intervals [lower (l-) and upper (u-) BCI and p-MCMC] of the four Linear Mixed Models (LMMs) fitted for the four behaviors studied in this study and the habitat characteristics of the center of the home range (Grain size and depth).

## Data accessibility

The data underlying this study are available from the Dryad Digital Repository (http://dx.doi.org/10.5061/dryad.q3c3t) [46].
