# Supplementary material for: Repeatability of circadian behavioural variation revealed in free-ranging marine fish
Source: R Soc Open Sci. 2017 Feb 15;4(2):160791. doi: 10.1098/rsos.160791 (PMC5367275; doi:10.1098/rsos.160791)
Supplement: Figure S1 Map of the study area and array of acoustic omnidirectional receivers where individuals of free-ranging peraly razorfish, Xyrchthys novacula, were electronically tracked [file rsos160791supp1.pdf]

Supplementary material for the manuscript entitled:

Repeatability of circadian behavioural variation revealed in free-ranging marine fish

By:

Josep Alós*, Martina Martorell and Andrea Campos-Candela

*Instituto Mediterráneo de Estudios Avanzados, IMEDEA (CSIC-UIB). C/ Miquel Marqués 21, 07190, Esporles, Illes Balears, Spain
E-mail: alos@imedea.uib-csic.es

Content:

Figure S1 Map of the study area and array of acoustic omnidirectional receivers where individuals of free-ranging peraly razorfish, *Xyrchthys novacula*, were electronically tracked.

Figure S1 Map of the study area and array of acoustic omnidirectional receivers where individuals of free-ranging peraly razorfish, *Xyrchthys novacula*, were electronically tracked. (A) location (black star) of the marine protected area (MPA) of Palma bay located in Mallorca Island, NW Mediterranean sea. (B) location of the array of 21 omni-directional acoustic receivers (black points) located in the MPA. The detection range where the ca. 50% are detected (300 m) is plotted as an empty grey circle around each receivers (see Alós et al. 2016 the detailed detection probability curve against distance). The habitat of the study area was composed by seagrass of *Posidonia oceanica* (PO), photophilic algae habitats (PA), fine-grain sand (FGS), medium-grain sand (LGS) and large-grain sand (LGS). Note how the suitable habitat for the pearly razorfish (FGS and MGS) is surrounded by PO restricting the movement within this area and limiting dispersal. Note how longitude is negatively correlated with depth, i.e., moving western, moving deeper, and latitude is negatively correlated with fine sand (grain size or habitat). (C) picture of one omni-directional acoustic receiver (Sonotronics© model SUR-1).

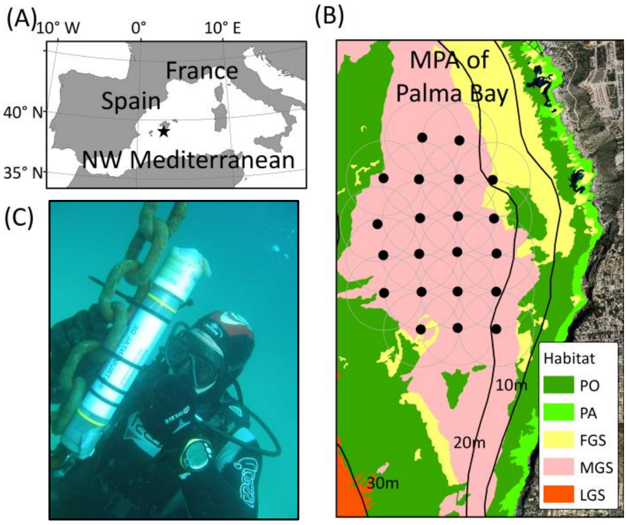

References cited in the Supplementary material:

Alós J, Palmer M, Balle S, Arlinghaus R (2016) Bayesian state-space modelling of conventional acoustic tracking provides accurate descriptors of home range behavior in a small-bodied coastal fish species. PLoS ONE 11:e0154089
