# Supplementary material for: Repeatability of circadian behavioural variation revealed in free-ranging marine fish
Source: R Soc Open Sci. 2017 Feb 15;4(2):160791. doi: 10.1098/rsos.160791 (PMC5367275; doi:10.1098/rsos.160791)
Supplement: Movie S1 Underwater video of free-ranging pearly razorfish, Xyrithchys novacula in the waters of Mallorca Island [file rsos160791supp2.pdf]

Supplementary material for the manuscript entitled:

Repeatability of circadian behavioural variation revealed in free-ranging marine fish

By:

Josep Alós*, Martina Martorell and Andrea Campos-Candela

*Instituto Mediterráneo de Estudios Avanzados, IMEDEA (CSIC-UIB). C/ Miquel Marqués 21, 07190, Esporles, Illes Balears, Spain
E-mail: alos@imedea.uib-csic.es

Content:

Movie S1 Underwater video of free-ranging pearly razorfish, *Xyrithchys novacula* in the waters of Mallorca Island.

Movie S1 High quality underwater video of the species case-study. The movie shows individuals of free-ranging pearly razorfish, *Xyrithchys novacula* in the waters of Mallorca Island (NW Mediterranean). The movie shows the characteristic burring behaviour of this species moving inside and outside the sand following a circadian rhythm. *X. novacula* is a protogynous hermaphrodite with marked sexual dimorphism and females (with a white spot in the body) and males (larger and coloured individuals) are identified in the movie. The video was filmed by Fernando Garfella for the publication after 50 h of scuba diving.

Link (Dropbox) to the video (only for reviewing process)*:
https://www.dropbox.com/s/4nl8ga1zdzfc1mc/MovieS1%20RoyalSocietyOpenScience.mp4?dl=0

**We will contact the relevant journal editorial office for further assistance for this large (i.e. larger than 10 MB) supplementary material according to author guidelines if the manuscript is accepted for publication*
