# Supplementary material for: Repeatability of circadian behavioural variation revealed in free-ranging marine fish
Source: R Soc Open Sci. 2017 Feb 15;4(2):160791. doi: 10.1098/rsos.160791 (PMC5367275; doi:10.1098/rsos.160791)
Supplement: Table S2 Environmental co-variates (posterior mean showed) and their Bayesian Credibility Intervals [lower (l-) and upper (u-) BCI and p-MCMC] of the four Linear Mixed Models (LMMs) fitted for the four behaviors studied in this study and the habitat characteristics of the center of the home range (G [file rsos160791supp3.pdf]

Supplementary material for the manuscript entitled:

Repeatability of circadian behavioural variation revealed in free-ranging marine fish

By:

Josep Alós*, Martina Martorell and Andrea Campos-Candela

*Instituto Mediterráneo de Estudios Avanzados, IMEDEA (CSIC-UIB). C/ Miquel Marqués 21, 07190, Esporles, Illes Balears, Spain
E-mail: alos@imedea.uib-csic.es

Content:

Table S1 Individuals of free-ranging pearly razorfish, *Xyrithchys novacula*, acoustically tracked in the marine protected area of Palma bay (NW Mediterranean).

Table S2 Environmental co-variates (posterior mean showed) and their Bayesian Credibility Intervals [lower (l-) and upper (u-) BCI and p-MCMC] of the four Linear Mixed Models (LMMs) fitted for the four behaviors studied in this study and the habitat characteristics of the center of the home range (Grain size and depth).

Table S1 Individuals of free-ranging pearly razorfish, *Xyrithchys novacula*, acoustically tracked in the marine protected area of Palma bay (NW Mediterranean). The identification code of the individual (id), the year of tagging, the fish size (in total length nearest the mm) and gender of the individuals, the first date and number of days (*n*) of tracking and the total number of detection by each individual are shown along the individual mean and s.d. of the four behaviors studied: awakening time relative to sunrise, rest onset related to sunset, rest duration and daily travelled distance.

							Awakening time (min)		Rest onset (min)		Rest duration (h)		Travelled distance (m)	
Id	Year	Fish size (mm)	Gender	First day	*n*	Acoustic detections	mean	s.d.	mean	s.d.	mean	s.d.	mean	s.d.
201102	2011	187	Male	02/08/2011	13	28,596	100.2	49.4	8.4	3.0	11.7	0.8	440.7	60.3
201104	2011	209	Male	01/08/2011	14	39,004	81.4	59.1	0.1	6.9	11.4	0.9	557.2	144.6
201107	2011	185	Male	01/08/2011	14	21,889	211.1	105.2	11.9	3.1	13.4	1.8	490.2	147.4
201109	2011	158	Female	03/08/2011	9	7,282	114.9	140.0	-8.7	8.6	11.2	0.5	310.5	92.3
201111	2011	159	Female	01/08/2011	14	48,577	29.3	13.3	5.8	4.0	10.4	0.2	376.5	115.7
201113	2011	192	Male	01/08/2011	14	54,242	18.2	18.1	-3.5	9.7	10.4	0.4	402.2	95.1
201202	2012	160	Female	21/08/2012	10	14,344	118.3	33.1	7.5	5.1	12.5	0.6	830.7	155.1
201204	2012	190	Male	19/08/2012	12	13,331	130.4	32.4	3.6	6.5	12.8	0.4	1073.4	287.4
201205	2012	220	Male	18/08/2012	12	28,121	74.7	27.0	5.1	5.0	11.5	1.3	135.4	33.6
201206	2012	179	Male	27/08/2012	4	4,041	211.8	16.2	7.0	1.6	14.5	0.5	1058.7	142.9
201208	2012	176	Male	21/08/2012	3	5,075	271.0	39.9	-9.3	4.6	15.1	0.4	657.2	182.2
201211	2012	185	Male	18/08/2012	10	8,940	192.0	56.0	13.4	3.3	13.8	0.8	1340.2	242.1
201213	2012	182	Male	19/08/2012	12	19,432	144.6	33.2	11.1	3.0	12.8	0.6	366.2	74.8
201219	2012	162	Female	24/08/2012	7	6,811	194.1	14.3	9.4	3.1	13.8	0.2	183.6	44.4

Table S2 Environmental co-variates (posterior mean showed) and their Bayesian Credibility Intervals [lower (l-) and upper (u-) BCI] of the four Linear Mixed Models (LMMs) fitted for the four behaviours studied here. The table shows the LMMs after the reduction according to the maximum explanatory power using the Deviance Information Criterion (DIC). Grain size and depth refer the habitat in the centre of the home range and were considered continuous variables, and year of experimentation was treated as categorical variable (showed the estimated of 2012 with respect 2011). The between- (V_{e_0}) and within-individuals (V_{e_0}) variances as well as the adjusted repeatability (adjusted-*R*) for each trait are also shown. The DIC of the reduced LMM as well as the DIC of the constrained LMM (DICc) are shown for all behavioural traits.

Awakening time (min)	Mean	l-BCI	u-BCI
Intercept	80.74	29.19	125.75
Grain size (Habitat)	34.51	2.31	64.15
Year (2012)	89	17.13	149.41
V_{ind_0}	2239	881.9	7220
V_{e_0}	3187	2488	4090
Adjusted-*R*	0.41	0.26	0.63
DIC = 1252 (DICc= 1824)			
**Rest onset (min)**	**Mean**	**l-BCI**	**u-BCI**
Intercept	4.73	1.75	7.71
Depth (m)	-4.09	-6.64	-1.3
V_{ind_0}	17.9.49	9.1	58.9
V_{e_0}	30.4	24.5	38.4
Adjusted-*R*	0.37	0.27	0.6
DIC = 1012 (DICc = 1079)			
**log-Rest duration (h)**	**Mean**	**l-BCI**	**u-BCI**
Intercept	2.41	2.34	2.48
Grain size (Habitat)	0.04	0	0.09
Year (2012)	0.18	0.09	0.28
V_{ind_0}	0.01	0.002	0.014
V_{e_0}	0.005	0.004	0.007
Adjusted-*R*	0.55	0.32	0.68
DIC = -338.2 (DICc = -259)			
**log-Travelled distance (m)**	**Mean**	**l-BCI**	**u-BCI**
Intercept	6.13	5.72	6.49
Grain size (Habitat)	0.25	-0.12	0.65
V_{ind_0}	0.34	0.12	0.94
V_{e_0}	0.07	0.05	0.08
Adjusted-*R*	0.82	0.77	0.91
DIC = 42 (DICc = 310)			
